# Stroke as a Paraneoplastic Manifestation of Ovarian Cancer: A Case Report

**DOI:** 10.7759/cureus.29835

**Published:** 2022-10-02

**Authors:** Rishman Tandi, Sourav Bansal, Sweta Sahu, Mummareddi Dinesh Eshwar, Prithvi Raghavan, Ojas V Kulkarni, Balaganesh Natarajan, Saikrishna Dodda, Anup Banur

**Affiliations:** 1 Medicine, Government Medical College, Amritsar, IND; 2 Surgery, Jagadguru Jayadeva Murugarajendra (JJM) Medical College, Davanagere, IND; 3 Biochemistry, Mahavir Institute of Medical Sciences, Hyderabad, IND; 4 Internal Medicine, Osmania Medical College, Hyderabad, IND; 5 Medicine, SMBT Institute of Medical Sciences (IMS) and Research Centre (RC), Nashik, IND; 6 Internal Medicine, St. George's University School of Medicine, St. George, GRD; 7 Medicine, Navodaya Medical College, Raichur, IND; 8 Pulmonology, SS Institute of Medical Sciences (IMS) and Research Centre (RC), Davanagere, IND

**Keywords:** microangiopathy, deep venous thrombi, stroke, paraneoplastic neurological syndromes, ovarian cancers

## Abstract

People with gynecologic neoplasms have the highest risk of having an ischemic stroke. A 76-year-old woman came into the stroke unit of our hospital complaining of anosmia and acutely developing dysarthria. She was ultimately determined to have ovarian cancer after extensive testing. Ovarian carcinoma is one of the neoplasms that cause ischemic stroke and is most commonly documented in case studies. Identifying the underlying neoplastic condition in female ischemic stroke patients who are otherwise "healthy" is crucial as an early surgical intervention on cancer offers therapeutic treatment for both malignancy and thromboembolism.

## Introduction

Deep venous thrombosis (DVT) is the most prevalent clinical manifestation of advanced cancer. Nevertheless, thrombotic arterial microangiopathy and/or systemic embolization may occur. A common consequence of advanced cancer is a prothrombotic or hypercoagulable condition, ranging between severe clinical thromboembolism and asymptomatic abnormal coagulation tests [1-2]. Because various cancer types produce distinct procoagulant chemicals or activate platelets, there might be a synergistic mechanism of thrombosis in cancer patients. Interleukin and tumor necrosis factor, which harm endothelial cells and make the surface of heart valves thrombogenic, may be released by malignant cells, which may result in the production of thrombin when combined with activated platelets and certain coagulation factors [3]. Early-illness symptoms are modest, vague, or non-existent. Because most patients get a diagnosis at an advanced stage, thrombosis may develop prior to ovarian cancer identification, and stroke may be the first clinical sign of this deadly illness [4]. Standard anticoagulation with heparin or a cumarine derivative does not help ovarian cancer patients with hypercoagulation, but it has been shown that removing the cancerous tumor stops it [5-6]. 

## Case presentation

The stroke unit of our hospital received a 76-year-old woman who reported a rapid onset of dysarthria and loss of smell. Examination revealed her blood pressure to be recorded to be 110/70 mmHg, pulse 77 beats per minute and regular, and temperature 36.9°C. A neurological examination revealed that the patient was conscious and oriented to time, place, and people. The Glasgow Coma Scale (GCS) was observed to be E4V3M6. She had bilateral anosmia and dysarthria. She had normal corneal and conjunctival reflexes and no deviation of the tongue. Power was noted to be 5/5, reflexes were 2+ in upper as well as lower limbs. Upon plantar reflex assessment, flexion of the big toe was observed which happened to be normal. Normal heart sounds S1 and S2 were heard and the lungs were clear on auscultation. There was no peripheral edema present. The patient was a known case of hypertension for five to six years and was well controlled with medication. The patient neither used alcohol nor did smoke. She had no notable history in her family, social circle, or environment.

Her past history revealed that four days earlier, the patient had presented with left homonymous hemianopia and expressive aphasia and was diagnosed with stroke. On MRI, ischemic infarction in the posterior cortical artery (PCA) territory was reported. After adequate workup and treatment, the patient was diagnosed with an ischemic infarction of an undetermined cause and discharged on antiplatelet drugs.

**Figure 1:**
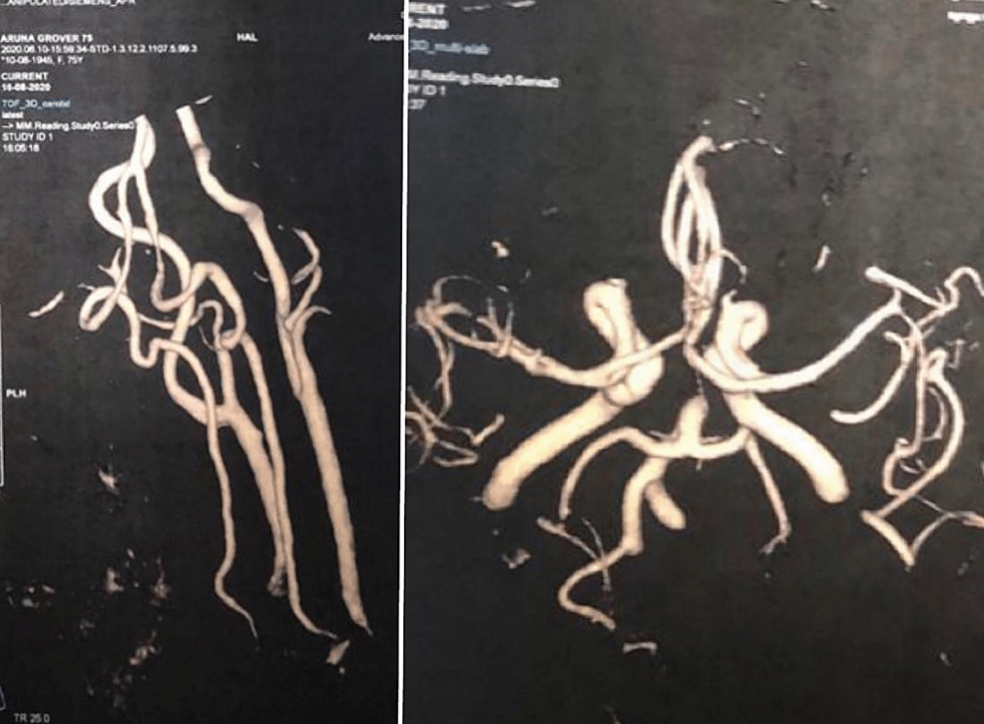
MRA of neck vessels (left) and circle of Willis (right). MRA, magnetic resonance angiography

Laboratory investigations were conducted which showed thrombocytopenia with a platelet count at 1,10,000, anemia with hemoglobin (Hb) at 9.4 g%, and a D-dimer level of 4.7 (normal <0.50). Diffusion-weighted MRI of the brain visualized triple territory non-hemorrhagic infarcts in the right PCA and the right and left middle cerebral arteries (MCAs), suggestive of embolic stroke of an undetermined source (ESUS). Investigations to assess vascular risk factors and secondary causes of stroke were conducted, which turned out to be normal. These included magnetic resonance angiography (MRA) of neck vessels and the circle of Willis, which showed completely normal vasculature with no occlusion (Figure 1). Further, transthoracic echocardiography was done to rule out any cardiogenic thrombosis. It showed that there was the presence of mild-moderate mitral regurgitation and the ejection fraction was 55% with no vegetation. Holter monitoring for 72 h was also normal.

Further imaging techniques included ultrasonography (USG) and MRI of the abdomen, which depicted a right ovarian mass lesion with multiple cystic and solid components. Her serum cancer antigen 125 (CA-125) came out to be greater than 100 (normal <35). The patient was transferred to the gynecology unit to further investigate ovarian pathology. A diagnosis of ovarian neoplasm was established. On a positron emission tomography (PET) scan, local spread to the uterus was detected but no distant metastasis was seen. Radical surgery was performed on the patient, including a total hysterectomy and bilateral salpingo-oophorectomy.

## Discussion

Stroke is among the most sequelae problems of cancer. The synergistic interaction between monocytes or macrophages and cancerous cells, which results in the release of tumor necrosis factor and interleukins-1 and -6, is the pathophysiology involved in this process. Injured endothelial cells act as a thrombogenic surface, which results in the activation of the blood coagulation cascade. Clots are formed when this cascade is activated. Fibrinolysis is not achieved in cancer patients as the tumor cells produce plasminogen inactivators that result in increased chances of developing a clot [3]. Cryptogenic strokes are fairly common, and they can offer the opportunity for screening for occult malignancy under the right circumstances [7].

The outlook for ovarian cancer, which is the third most common type of cancer in gynecologic tumors, is very negative [8]. The disease's early stages produce few, vague, or no symptoms [9]. As a result, a large group of patients receive diagnoses at a progressing stage; as a result, thrombosis can develop prior to the discovery of ovarian cancer, and stroke can be the initial clinical sign of this cancerous condition, as was the case in our patient [10]. Ischemic stroke is associated with many risk factors, including age over 50, diabetes mellitus, hypertension, and chemotherapy [11]. The D-dimer test is a biomarker that globally indicates the activation of hemostasis and fibrinolysis. Literature has proved that the levels of D-dimer in blood predict the incidence of stroke in cancer patients [12]. Our observation of elevated D-dimers supported by infarcts in the right MCA, PCA, and left MCA, along with clear vessels in the neck and circle of Willis on MRA, arose suspicion of cryptogenic stroke of an unknown source. Furthermore, cardioembolic pathology was excluded by doing transthoracic echocardiography of the patient. With a negative patient history and a diagnosis of ESUS at hand, PET scan ultrasound abdomen, MRI abdomen, and CA 125 proved the diagnosis of ovarian cancer. On excluding the possible causes of stroke, this cryptogenic stroke was most likely due to paraneoplastic disseminated intravascular coagulation (DIC) [13]. By definition, paraneoplastic syndrome refers to the distant effects of the malignancy that are unrelated to the site of tumor invasion or metastasis [14]. 

## Conclusions

Our case gave us a learning lesson that suspicion of cancer should always be kept in mind while treating a female with a stroke with no relevant history in order to detect underlying ovarian cancer. Early and meticulous diagnosis and surgical intervention will improve cancer treatment. Individuals receiving chemotherapy, particularly regimens including platinum-based medicines, and people over the age of 50 were all significant risk factors. Other risk factors included having an ancestral history of ovarian cancer. Patients with ovarian cancer are likely to live longer than patients with other types of cancer. This means that strokes may need to be prevented through regular monitoring and the use of effective interventions.
